# Ocular Complications of Obstructive Sleep Apnea

**DOI:** 10.3390/jcm10153422

**Published:** 2021-07-31

**Authors:** Pei-Kang Liu, Tzu-Yu Chiu, Nan-Kai Wang, Sarah R. Levi, Ming-Ju Tsai

**Affiliations:** 1 Department of Ophthalmology, Kaohsiung Medical University Hospital, Kaohsiung Medical University, Kaohsiung 807, Taiwan; aleckliu418@gmail.com (P.-K.L.); kathychiu77924@hotmail.com (T.-Y.C.); 2 School of Medicine, College of Medicine, Kaohsiung Medical University, Kaohsiung 807, Taiwan; 3 Institute of Biomedical Sciences, National Sun Yat-sen University, Kaohsiung 804, Taiwan; 4 Department of Ophthalmology, Edward S. Harkness Eye Institute, Columbia University, New York, NY 10032, USA; wang.nankai@gmail.com (N.-K.W.); Slevi@wesleyan.edu (S.R.L.); 5 Division of Pulmonary and Critical Care Medicine, Department of Internal Medicine, Kaohsiung Medical University Hospital, Kaohsiung Medical University, Kaohsiung 807, Taiwan; 6 Sleep Disorders Center, Kaohsiung Medical University Hospital, Kaohsiung Medical University, Kaohsiung 807, Taiwan; 7 Department of Respiratory Care, College of Medicine, Kaohsiung Medical University, Kaohsiung 807, Taiwan; 8 Graduate Institute of Clinical Medicine, College of Medicine, Kaohsiung Medical University, Kaohsiung 807, Taiwan

**Keywords:** obstructive sleep apnea, sleep-disordered breathing, nocturnal hypoxia, eye, complications, oxidative stress, inflammation, sympathetic activation

## Abstract

Obstructive sleep apnea (OSA), the most common form of sleep-disordered breathing, is characterized by repetitive episodes of paused breathing during sleep, which in turn induces transient nocturnal hypoxia and hypercapnia. The high prevalence of OSA and its associated health consequences place a heavy burden on the healthcare system. In particular, the consequent episodic oxygenic desaturation/reoxygenation series and arousals from sleep in patients with OSA have the potential to trigger oxidative stress, elevated systemic inflammatory responses, and autonomic dysfunction with sympathetic activation. Given these adverse side-effects, OSA is highly correlated to many eye diseases that are common in everyday ophthalmic practices. Some of these ocular consequences are reversible, but they may permanently threaten a patient’s vision if not treated appropriately. Here, this article seeks to review the ocular consequences and potential pathophysiologic associations in patients with OSA. Understanding these OSA-related eye diseases may help clinicians provide comprehensive care to their patients.

## 1. Introduction

Sleep apnea (SA), the most common form of sleep-disordered breathing, causes recurrent pauses in breathing during sleep and transient nocturnal hypoxia and hypercapnia [1,2,3]. The diagnosis of SA is usually confirmed by polysomnography (PSG) [4,5]. Obstructive SA (OSA) is the most common type, accounting for over 85% of all SA cases, whereas central SA (CSA) is a rarer presentation [6]. OSA is characterized by repetitive bouts of complete or partial upper airway obstruction during sleep, which induces loud snoring and airflow reduction [7,8]. These episodic oxygenic desaturation/reoxygenation series and arousals from sleep/sleep fragmentation in patients with OSA have severe consequences, including oxidative stress, autonomic dysfunction with sympathetic activation, blood pressure spikes, increased heart rate, insulin resistance, enhanced platelet aggregation, endothelial dysfunction, and heightened systemic inflammation [9,10,11,12,13,14,15,16,17,18,19]. The Apnea–Hypopnea Index (AHI, total number of apnea and hypopnea events divided by total sleep time) is usually used to determine the severity of OSA, classifying patients into four categories: subjects without OSA have an AHI of <5/h, patients with mild OSA have an AHI of 5–15/h, patients with moderate OSA have an AHI of 15–30/h, and patients with severe OSA have an AHI of >30/h [20]. As an emerging health issue in the contemporary world, OSA is estimated to affect 22–24% of men and 9–17% of women globally, while it is also associated with an overweight body mass index (BMI) [21,22].

With all the adverse effects associated with OSA, its secondary effects cause a number of ocular complications [16,23,24]. Previous studies have shown that OSA is associated with increased risks of several vision-threatening and nonthreatening ocular disorders, including senile cataract, normal tension glaucoma, retinal ischemia, conjunctival hyperemia, and dry eye [23]. Several contributory mechanisms to the ocular complications of OSA have been reported, including intermittent hypoxia, oxidative stress, systemic inflammatory responses (such as interleukin-6 (IL-6), IL-8,tumor necrosis factor-alpha (TNF-α), C-Reactive protein (CRP), matrix metalloproteinase 9 (MMP-9), vascular cell adhesion molecule (VCAM), intercellular adhesion molecule (ICAM), selectins), sympathetic system overaction, damage effects of endothelin-1, and disruption of the blood–retinal barrier (BRB) (Table 1) [11,12,13,14,15,16,18,19,24,25,26]. In addition, OSA-mediated chronic upregulation of systemic inflammation may provoke both ocular and non-ocular complications, such as psoriasis and rheumatoid arthritis, which may cause further ocular abnormalities [27,28].

**Figure 1:**
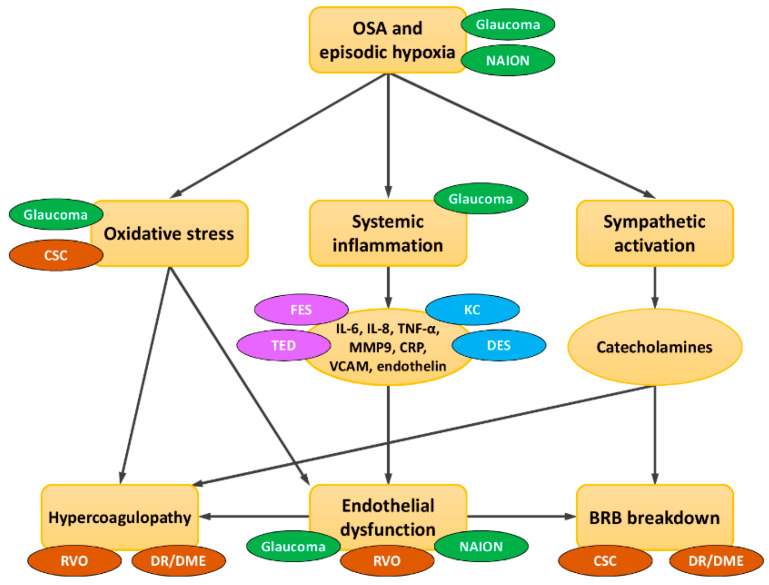
Schematic interactions of OSA and eye diseases in terms of common pathophysiological mechanisms. OSA has been directly/indirectly linked to many ocular diseases through three major mechanisms: oxidative stress, elevated systemic inflammation, and sympathetic activation. In glaucoma, hypoxia/hypercarbia directly damages high oxygen-depleted neuronal cells and triggers systemic inflammation and oxidative stress, leading to mitochondrial dysfunction and provoking ganglion cell apoptosis. Glaucoma is also directly aggravated by the mechanical damage caused by increased nocturnal intraocular pressure. In non-arteritic anterior ischemic optic neuropathy (NAION), nocturnal hypoxemia and impaired vascular autoregulation of posterior ciliary arteries may directly predispose NAION. Thyroid eye disease (TED), floppy eyelid syndrome (FES), central serous chorioretinopathy (CSC), dry eye syndrome (DES), and keratoconus (KC) may share some common excessive circulating cytokines and tissue inflammatory mediators with OSA. Hypercoagulopathy in OSA may contribute to retinal vascular occlusion (RVO) and diabetic retinopathy (DR)/diabetic macular edema (DME). OSA-induced vascular endothelial dysfunction and narrowing of blood vessels may lead to ischemia and damage to the delicate vessels in the optic nerve head and retina. Blood–retinal barrier (BRB) breakdown predisposes CSC and DR/DME. An increase in systemic oxidative stress may also induce the development of CSC. green = optic nerve; brown = posterior segment; purple = orbit and eye lid; blue = anterior segment.

Due to the prevalence of OSA in the working age population, an understanding of the ocular conditions associated with OSA and their underlying pathophysiological mechanisms is essential for the comprehensive care of patients. Herein, we review the ocular consequences and associated underlying pathophysiological mechanisms in patients with SA (Figure 1). Ultimately, this article aims to assist clinicians in understanding and diagnosing ocular conditions in patients with OSA in the hope of preventing future irreversible eye complications.

## 2. OSA-Associated Complications in Optic Nerve

### 2.1. OSA and Glaucoma

Glaucoma is a group of progressive optic disorders characterized by structural and corresponding functional abnormalities. Currently, glaucoma is the second leading cause of blindness in the world and is estimated to affect 111.8 million people by the year 2040 [29]. Elevated intraocular pressure (IOP) is the major modifiable risk factor for this optic disorder; however, glaucoma can exist or even progress when IOP is measured within a normal range. In this case, it is critical to consider other systemic diseases, such as hypotension or OSA, as a potential cause of glaucoma.

The relationship between OSA and normal tension glaucoma (NTG) and/or primary open angle glaucoma has been discussed in many contrasting studies (Table A1 in Appendix A). Some studies showed a higher prevalence of glaucoma in OSA patients; thus, routine OSA screening is recommended [30,31,32,33]. Conversely, there has been no difference in the reported prevalence of glaucoma between OSA and non-OSA populations [34,35]. Compared to non-OSA, several studies revealed higher IOP values in OSA patients [30], while some other studies showed no difference [36]. In 2016, Huon et al. performed a meta-analysis of six case–control studies analyzing the prevalence of OSA among patients with glaucoma and 10 cross-sectional plus two case–control studies on the prevalence of glaucoma among patients with OSA, revealing the increased odds of both glaucoma diagnosis among OSA patients (odds ratio (OR): 1.242, *p* < 0.001) and OSA diagnosis in glaucoma patients (OR: 1.746, *p* = 0.002) [37].

Anatomically, a thinner retinal nerve fiber layer (RNFL) [38,39,40], lower ganglion cell complex thickness [41,42], lower peripapillary vessel density, and increased foveal avascular zone in optical coherence tomography angiography [43,44] are all reported in patients with greater OSA severity. Functionally, a higher incidence of visual field defects, which correlate with AHI in NTG [45], as well as an amplitude reduction and longer latency period in visual evoked potential (VEP), has been reported [46,47].

The precise etiological mechanisms underlying OSA-mediated glaucoma remain unclear, but there are several possible explanations. One theory suggests “mechanical” damage due to nocturnal increased IOP from supine sleep posture, altered sleep hormone balance (a nocturnal serum melatonin peak, which is associated with lowered IOP during sleep, was absent in OSA patients), obesity with excessive intraorbital adipose tissue or increased episcleral venous pressure, and autonomic dysfunction [48,49,50,51,52,53,54,55]. Another theory explores ischemia or abnormal perfusion as the cause of optic disc vulnerability leading to the observed normal IOP value [38]. A third theory points to a “vascular dysregulation” event of the optic nerve. OSA is associated with several vascular diseases. It causes changes in the vascular endothelium, narrowing of blood vessels, reduced availability of nitric oxide (a vasodilator), and increased levels of endothelin-1 (a vasoconstrictor), leading to ischemia and damage to the optic nerve head [50]. Another theory presumes that episodes of “hypoxia/hypercarbia” directly damage high oxygen-depleted neuronal cells and trigger systemic inflammation and oxidative stress, leading to mitochondrial dysfunction and ganglion cell apoptosis [56]. High-tension glaucoma or ocular hypertension shares risk factors and comorbidities with high AHI, which may be a confounding factor for the higher incidence of glaucoma in the OSA group [57].

In addition to the standard treatment of glaucoma, there is limited strategy available to specifically treat OSA-related glaucoma. The available evidence on continuous positive airway pressure (CPAP) for treatment is controversial. One study reported that higher diurnal variations of IOP occurred in OSA patients after CPAP therapy, which may have an effect on glaucoma progression [58]. More recent studies have shown no difference in nocturnal IOP elevation between CPAP and non-CPAP groups [34,59]. In 2019, Lin et al. published a prospective study showing significant improvement of visual sensitivity, RNFL thickness, and macular layer thickness in OSA patients after 3 months of CPAP treatment with good adherence [60]. Due to the controversial safety of CPAP therapy on glaucomatous patients, long-term ophthalmic monitoring and further randomized controlled trials are required.

### 2.2. OSA and Ischemic Optic Neuropathy

Non-arteritic anterior ischemic optic neuropathy (NAION), a microvascular infarction of the anterior portion of the optic nerve, presents as a sudden, painless, usually unilateral and irreversible vision loss, which frequently attacks upon awakening. NAION typically occurs in middle-aged adults and has a strong link to conditions with risks of optic nerve hypoperfusion, including hypertension, cigarette smoking, diabetes mellitus (DM), ischemic heart disease, crowded disc, and dyslipidemia. Clinical signs include a relative afferent pupillary defect, papilledema, flame shape hemorrhage, and consequent optic atrophy [61].

The association between NAION and OSA has been discussed in many studies (Table A2 in Appendix A). In 1988, Bucci et al. first described a 46 year old male with OSA and optic disc edema [62]. More recently, Huon et al. published a meta-analysis of four case–control studies showing that OSA was significantly associated with NAION development (pooled OR: 3.126, *p* < 0.001) [37]. Another meta-analysis showed that the pooled OR of developing NAION in OSA group was 6.18 compared with controls (*p* = 0.002) [37]. Stein et al. also assessed the significantly increased hazard of having NAION in an OSA cohort [34]. A large retrospective longitudinal cohort study using the Taiwan National Health Insurance Database, including 8488 patients of SA and 33,952 control subjects, showed that SA was a significant risk factor for the development of NAION (hazard ratio (HR): 1.66) [63]. In addition, in OSA patients without medical comorbidities, VEP showed a reduced amplitude and longer latency of the P100 component compared to healthy controls [46]. Although the exact mechanism via which OSA induces NAION is unknown, several hypotheses have been proposed. Specifically, the impaired vascular regulation within the optic nerve may result from interaction of the following: direct nocturnal hypoxemia, endothelial dysfunction and altered autoregulation of posterior ciliary arteries from intermittent sympathetic surges and oxidative stress, imbalance between vasoactive sub-stances (such as nitric oxide and endothelin), abnormal platelet aggregation, and/or decreased cerebral perfusion pressure from hypoxia-induced cerebral vasodilation [64,65]. The intracranial pressure elevation from apneic events may also directly damage the optic nerve or indirectly affect the optic nerve via compromising circulation [64,65].

Considering the role of CPAP in the treatment of OSA, a few studies have reported the protective effect of CPAP in minimizing the development of NAION [34,66]. Poor compliance with CPAP therapy, however, was a major risk factor of NAION in the fellow eye [66]. On the other hand, stringently adherent CPAP therapy also significantly improved VEP in patients with OSA [67]. Given the strong association between OSA and NAION, PSG may be suggested in patients with newly diagnosed NAION, especially in younger patients, as well as those without other risk factors [68].

## 3. OSA-Associated Complications in Orbit and Eyelids

### 3.1. OSA and Thyroid Eye Disease (TED)

Thyroid eye disease (TED), also known as Graves’ ophthalmopathy, is an inflammatory, autoimmune, and biphasic disorder that presents with variable degrees of exophthalmos, lid retraction, strabismus, congestive orbital signs, and compressive optic neuropathy [69]. TED mostly occurs in patients with Graves’ hyperthyroidism but can also be seen in Hashimoto’s thyroiditis or euthyroid [69,70]. The pathogenesis of TED is not completely characterized. However, it is known that the orbital fibroblasts, activated by thyroid-stimulating hormone (TSH) and insulin-like growth factor 1, play a key role in modulating the inflammatory process and causing pathologic accumulation of glycosaminoglycans, fibrosis, and adipogenesis in orbital soft tissues [69,70,71].

The association between SA and thyroid hormone alteration has been discovered since the 1980s (Table A3 in Appendix A) [72,73]. Bruyneel et al. assessed the higher prevalence of thyroid disorders in the OSA population as compared to the general population, with a particular focus on patients with higher BMI values [74]. Within the last decade, Bozkurt et al. reported that OSA patients presented with higher prevalence of Hashimoto’s thyroiditis in conjunction with higher OSA severity, especially among women [75]. Specifically, there is in fact a significant correlation between the mean apnea duration and free triiodothyronine (fT3) level; individuals with lower fT3 (≤3.75 pg/mL) had longer mean apnea time as compared to those with higher fT3 (>3.75 pg/mL) [76]. Patients with hypothyroidism are also at higher risk for secondary sleep-disordered breathing, which could be OSA, CSA, or mixed SA [77].

The common mechanisms between OSA and hypothyroidism include macroglossia, deposition of mucopolysaccharides in the upper airway, disturbances of upper airway musculature, and suppression of the respiratory center [76]. With regard to complex inflammatory processes, OSA and TED may share some common circulating cytokines and tissue inflammatory mediators [18,25,71,78].

A retrospective study of 109 individuals demonstrated that the prevalence of higher-risk OSA in patients with compressive optic neuropathy from TED was significantly higher than in those without TED-related compressive optic neuropathy [78]. Another prospective observational cohort study observing 85 patients also revealed that the risk of compressive optic neuropathy and vertical strabismus from TED was significantly higher in patients with a higher risk of OSA [79]. In terms of the effects from CPAP, one report demonstrated normalization of fT3 after CPAP treatment in OSA patients with euthyroid sick syndrome [80]. Another randomized controlled study showed that TSH decreased after CPAP without changes in free T4 level [81]. Some studies also indicated that CPAP treatment is able to reduce serum levels of systemic inflammatory cytokines [19,82]. Given that smoking is the only known modifiable risk factor in TED, effective treatment of OSA may be another manageable risk factor for TED [78].

### 3.2. OSA and Floppy Eyelid Syndrome (FES)

Floppy eyelid syndrome (FES) was first described by Culberston and Ostler in 1981. This palpebral laxity disorder with papillary tarsal conjunctivitis and lax superior eyelids is characterized by flaccid upper eyelid tone, eyelash ptosis and easily everted eyelids (leading to ocular irritation), redness, and mucus discharge that worsen upon awakening [83]. The prevalence of FES among adult individuals has ranged from 3.8% to 15.8% in previous studies [84].

The association between FES and OSA has been discussed in many studies [85,86,87], identifying a positive correlation between OSA severity and FES. Wang et al. reported that the risk of FES is 4.12-fold higher in patients with OSA compared to the non-OSA population [88]. The exact pathogenesis of FES has not been clearly determined. Histological examination has demonstrated a marked loss of elastin fiber in tarsus and chronic inflammation of conjunctival epithelium [89]. One theory points to a direct mechanical trauma, prone posture during sleep, with palpebral and conjunctiva rubbing to the pillow resulting in super-expression of elastolytic enzymes and elastin degradation [90]. The other theory proposes activation of cytokines (TNF-α, MMP enzymes) from systemic inflammation and intermittent hypoxia related to OSA, leading to elastin breakdown [18,26,91].

FES patients with mild abnormalities may improve with conservative treatment, such as aggressive lubrication, eye shield/lid tapping at nighttime, and maintaining proper posture during sleep. In severe cases, further corrective surgery is frequently required. Although CPAP may have some negative effects due to air leakage from the mask edges [92], one case report described the resolution of ocular discomfort in patients with FES and OSA following CPAP use [93]. Another case report even found FES to be reversible after 4 years of CPAP treatment [94]. A prospective double-blind study evaluating the effects of CPAP showed that long-term (at least 1 year) use of CPAP improved the clinical manifestations of FES [95].

## 4. OSA-Associated Complications in Anterior Segment of Eye

### 4.1. OSA and Dry Eye Syndrome (DES)

Dry eye syndrome (DES), the most common ocular surface disorder throughout the world, is a multifactorial disease with a wildly varying prevalence from 5% to 50% [96]. The lacrimal functional unit consists of the cornea, conjunctiva, lacrimal glands, Meibomian gland, eyelid, and the sensory/motor nerves that connect them. Dysfunction in any of these components can lead to reduced tear production, increased tear osmolarity, and tear film layer instability that has been attributed to the etiologies of DES [97].

OSA is related to a decreased Schirmer value, which suggests a tendency toward aqueous deficient DES [98]. Shortened tear break-up time (TBUT) and noninvasive tear break-out time (NTBUT), which represent excessive tear evaporation, were also detected in patients with OSA [98,99,100]. Among patients using CPAP or other nasal mask therapies, the prevalence and incidence of DES were significantly higher, as compared to the general population [101]. Additionally, in patients with severe OSA, morphological alterations of the Meibomian glands, including ductal thinning, dilatation, and distortion, were more frequently demonstrated on meibography [102]. In a case–control study, patients with OSA showed greater loss of Meibomian glands in the upper and lower eyelids, as compared to controls, which significantly correlated with the severity of OSA [100].

Inflammation of the lacrimal gland and ocular surface plays the key role in the pathogenesis of dry eye, which typically develops as a result of high tear osmolarity, accumulation of proinflammatory cytokines from the lacrimal glands on eye surface, and delayed washing out by less tear production [103]. Sleep deprivation can interfere with the lacrimal system and subsequently induce DES, as supported in an experimental mouse model [104]. The levels of proinflammatory cytokines, such as IL-6, IL-8, and TNF-α, were elevated in patients with OSA [18,19,25]. Moreover, the cytokines were released from dilated conjunctival vessels and damaged epithelial cells, triggering continuous inflammation on the ocular surface, leading to Meibomian and goblet cell dysfunction and tear film deterioration [98].

In terms of the effects of CPAP treatment on the tear film, conflicting results have been presented in the literature. OSA patients undergoing CPAP therapy may encounter lower TUBT values and ocular complications such as dryness and irritation due to air leakage from the mask edge or mask displacement [105]. On the other hand, a few studies comparing OSA patients with or without CPAP treatment have shown that CPAP users had lower rates of abnormal eye irritation and TBUT values, attributed to appropriate, long-term CPAP treatment, as well as good control of systemic conditions [92,95].

### 4.2. OSA and Keratoconus (KC)

Keratoconus (KC), an ectatic disorder, is characterized by a progressive, bilateral but asymmetric cone-shaped cornea with central or paracentral thinning and protrusion. Genetic predisposition and environmental risk factors including eye-rubbing, atopy, inflammation, and contact lens wear have all been implicated in its etiology. Currently, the estimated prevalence of KC in the general population is approximately 54 per 100,000 [106].

In recent years, the relationship between KC and OSA has been increasingly studied, showing inconsistent results. A meta-analysis of one cohort and four case–control studies showed a significant association between KC and OSA (pooled OR: 1.841, *p* = 0.009) [107]. See et al. conducted a nationwide population-based matched case–control study and found no significant association between KC and SA [108]. Topographic and biomechanical corneal variations (corneal volume, corneal elevation, and minimum radius) [109], as well as greater pleomorphism and polymegathism of the corneal endothelium [110], were found in severe OSA when compared to healthy subjects. The percentage of rapid eye movement sleep, which helped corneal oxygenation and usually decreased in patients with OSA, was also negatively correlated with central corneal thickness [110].

The pathophysiology between KC and OSA is not clearly defined, and several possible mechanisms have been proposed. MMPs, a family of 24 zinc-dependent proteases with the ability to degrade collagen and other extracellular matrix (ECM) proteins in response to stress or injury, may play an important role in KC formation [111]. Patients with KC have higher MMP-9 in their tear component and serum [112,113]. Similarly, serum concentration of MMP-9 is elevated in patients with OSA and is related to OSA severity, inflammation, and/or severity of hypoxemia [26,114].

### 4.3. SA and Cataract

Cataracts affect individuals worldwide and represent one of the leading causes of visual disability, especially in developing countries [115,116,117,118,119]. In a recent small, cross-sectional case–control study, it was reported that patients with OSA were at greater risk of developing cataracts [23]. At this point in time, however, more evidence is still needed to confirm the relationship between cataracts and SA.

## 5. OSA-Associated Complications in Posterior Segment of Eye

### 5.1. OSA and Retinal Vein Occlusion (RVO)

The retina has a high oxygen demand and is one of the most metabolically active tissues in the body. As such, at a relatively early stage of the disease, the retina often exhibits changes secondary to hypoxic disease such as OSA [120]. Retinal vein occlusion (RVO) is the second most common vision-threatening retinal vascular disorder worldwide, following diabetic retinopathy [121]. The pathophysiology of RVO may relate to the principles of Virchow’s triad (hypercoagulability, blood stasis, and endothelial damage) that contribute to thrombosis formation [122,123,124]. OSA is known to impair endothelial function and induce hemodynamic changes. OSA may also increase hypercoagulability status through many mechanisms, such as proinflammation, oxidative stress, increased sympathetic activation from morning epinephrine surges, and excessive platelet activation [10,18,24,125,126,127,128,129]. The severity of OSA is associated with a reduced retinal arteriovenous ratio and diminished retinal vascular pulse amplitude, increasing vascular occlusion [130]. Nocturnal hypoxic events may consequently increase the risk of RVO via combination of Virchow’s triad during recurrent apneic events.

The association between OSA and RVO has been investigated in several studies (Table A4 in Appendix A). In a nationwide population-based study in Taiwan including 5965 sleep apnea patients and 29,669 control subjects, the researchers found that SA was an independent risk factor for RVO, bringing 1.94-fold increase in the incidence of RVO [131]. A prospective study systematically screening RVO patients and controls using a sleep evaluation revealed a significantly higher prevalence of OSA in RVO patients compared to controls, although no significant relationship between the degree of OSA and RVO was noted [132]. In an age and sex-matched control study, mean AHI and oxygen desaturation index (ODI) were significantly higher in patients with central RVO (CRVO). A positive correlation between AHI and BMI was also identified [133]. A study conducted by Brent et al. screened their 27 newly diagnosed RVO patients using PSG and revealed a high prevalence (up to 96%) of severe OSA [134]. This remarkable finding led to a mandatory screening for OSA in all patients with RVO. A recent case–control study reported that the oxidative stress factors and inflammatory biomarkers were elevated in serum from patients with OSA and RVO, and serum levels of oxidative/inflammatory biomarkers were positively correlated with the severity of OSA; OSA was a negative prognostic factor of visual activity improvement in patients with RVO [135]. Today, evidence for the effect of CPAP therapy on RVO is still lacking.

### 5.2. OSA and Central Serous Chorioretinopathy (CSC)

Central serous chorioretinopathy (CSC), characterized by localized serous detachment of the neurosensory retina and/or RPE mostly at the macula, frequently affects men under the age of 50 [3,136,137]. Elevated serum cortisol and catecholamine levels are known risk factors for CSC [11]. The intermittent episodes of hypoxia, i.e., physical stress after sudden arousal from sleep, can lead to increased sympathoadrenal activity and increased basal levels of circulating endogenous catecholamines, including epinephrine and norepinephrine, and the breakdown of the BRB in SA patients [11,12,13,14,15,16]. Increased systemic oxidative stress may be related to occurrence of CSC, which is one of the possible mechanisms via which OSA promotes the development of CSC [138].

Many studies have debated the association between SA and CSC (Table A5 in Appendix A). A few have favored a positive correlation between CSC and SA/OSA [11,12,37,139]. For example, a systemic meta-analysis found that CSC patients have a higher risk for OSA (OR: 1.56) [139]. However, a case–control study revealed that patients of CSC did not have significantly higher rates of OSA risk or previous diagnosis [140]. The conflicting results across studies may be biased by matching controls with BMI, a well-known risk factor for OSA [21].

A case–control study recruiting 183 consecutive CSC patients and 183 age-matched controls demonstrated that patients with OSA confer a significantly higher risk for developing CSC (OR: 4.97) [141]. A large-scale population-based cohort study in Taiwan, consisting of 10,753 patients with SA and 322,590 age- and sex-matched control subjects, focused on identifying the effect of SA on the incidence of CSC [3]. The study concluded that patients with SA had a significantly increased incidence rate of CSC vis-à-vis matched controls (incidence rate ratio (IRR): 1.2) after adjusting for sex, age, residency, income level, and comorbidities. This study also found that SA patients receiving CPAP titration had a significantly lower incidence rate of CSC than the other patients [3]. Another large-scale cohort study from United States had similar results, revealing a higher incidence (HR: 1.081) of CSC in patients with OSA [142]. Given that CSC is a reversible disease, CSC patients with an underlying OSA may benefit from the treatment of OSA, especially in patients with chronic or refractory CSC [3,143].

### 5.3. OSA and Diabetic Retinopathy (DR) and Diabetic Macular Edema (DME)

Diabetic retinopathy (DR) is a microvascular complication that is currently the leading cause of blindness in working-aged adults [144]. Classically well-recognized risk factors for DR are hyperglycemia, systolic blood pressure, and duration of diabetes [145]. Molecular mechanisms for the emergence of DR include increased oxidative stress and inflammation associated with the activation of a number of factors including vascular endothelial growth factor (VEGF), which causes a breakdown of the BRB [146,147]. The repeated hypoxemia–reoxygenation cycle of SA may instigate oxidative stress and systemic inflammatory responses and, therefore, may further aggravate the onset and progression of DR in patients with SA plus DR [9,148]. Recently, dysregulation of circadian locomotor output cycles kaput (CLOCK) genes was reported in animal models of OSA [149,150]. Expression alterations of specific circadian CLOCK genes (*CRY1* and *PER3*) may serve as independent predictors of OSA severity in patients [151]. Increased HIF-1α level is associated with the overexpression of circadian clock proteins and might mediate the development of circadian clock disruption in patients with OSA [152]. Upregulation of *DEC2*, regulated by the human CLOCK gene [153], upregulated retinal *HIF1α* and *VEGF* expression in human Müller glial cells under hypoxic conditions, which was frequently produced by OSA [154]. *VEGF* overexpression due to CLOCK gene dysregulation in SA patients may be involved in the common pathophysiology of DR progression in SA.

Many previous studies have disclosed that OSA is associated with development of DME, metabolic syndrome, increased hyperglycemia, and insulin resistance, as well as the onset and progression of DR, especially in the absence of treatment [155,156,157,158,159,160,161,162]. The connection between OSA and diabetes may be a two-way street (Table A6 and Table A7 in Appendix A). DM is also a risk factor for developing OSA. A large-scale retrospective cohort study of 1,656,739 patients revealed that the adjusted IRR of OSA in patients with type 2 DM was 1.48 (*p* < 0.001) compared to patients without type 2 DM. Obesity, male gender, and excessive BMI were the strongest predicting factors for OSA in individuals with type 2 DME (OR: 8.29, 2.27, and 2.02, respectively) [163].

A number of studies have investigated the correlation between DR and OSA severity, which was evaluated by AHI. Although a few studies did not find significant effects of OSA severity on DR [164,165], others demonstrated a positive relationship. In particular, a retrospective chart review in a veteran population reported the frequency of DR increases with the severity of OSA [166]. Patients with severe OSA were at increased risk of developing DR (OR: 2.18), proliferative DR (OR: 2.40), and DME (OR: 2.89) [167]. A cross-sectional study of 92 patients found that vision-threatening DR was associated with a higher AHI and higher risk of moderate OSA (OR: 1.06 and 4.73, respectively) [168]. Mori et al. demonstrated that AHI was independently associated with DR, and the OR of developing DR rose to 12.9 in patients with diabetes and OSA having AHI scores in the top quartile [169]. The 4% oxygen desaturation index (4% oxygen desaturation events per hour) may be a potential predictor of DR in patients with OSA. Another cross-sectional study of patients with type 2 DM showed that the 4% oxygen saturation index was significantly higher in the proliferative diabetic retinopathy (PDR) group than in the non-proliferative diabetic retinopathy (NPDR) group; the effect on patients with SA needs further confirmation [170].

DME is a multifactorial complication of altered BRB mainly from pathological overexpression of *VEGF* and is the leading cause of vision loss and reduced quality of life in developed countries across the world [171,172]. Patients with SA present with elevated levels of basal endogenous catecholamines, which consequently lead to endothelial dysfunction of the BRB [16]. Studies indicated that severe OSA with AHI over 30 and higher cumulative time of SpO_2_ less than 90% were associated with increased risk of developing DME [167,173]. OSA also increases the possibility of intractable DME. Patients who responded poorly to anti-VEGF therapy for DME were at significantly higher risk of having OSA [174,175]. A retrospective clinical cohort study using a database from Taiwan also suggested that severe OSA was a risk factor for DME and was involved in having refractory DME [175].

CPAP treatment may be beneficial for DR and DME. Some inflammatory markers (IL-6, IL-8, TNF-α) in serum were found to decrease significantly after 3 months of CPAP therapy [82]. A meta-analysis revealed that, after CPAP therapy, the level of VEGF, which motivated the progression of cardiovascular disease, was significantly reduced in OSA patients [176]. Mason et al. studied the effect of CPAP treatment on 35 patients with DME and OSA and found a possible association between CPAP usage and improvement in visual acuity after 6 months of treatment [177]. A later retrospective cross-sectional review found that patients with OSA who were compliant with CPAP therapy had a significantly lower risk of developing DR as compared to noncompliers (OR: 0.54) [178]. A prospective randomized controlled trial of 131 patients with DME and OSA revealed no increase in visual function gains over a 12 month period with CPAP for OSA compared to controls [179]. Although CPAP may not restore visual acuity from the existing DME in patients with OSA, it may be beneficial in reducing the development of DR/DME. Moving forward, more studies are needed to confirm the benefits of CPAP treatment on DR and DME in SA patients.

## 6. Discussion

As the global prevalence of obesity continues to increase, the prevalence of OSA, which is highly associated with BMI, can be expected to rise [180,181,182]. However, the rate of OSA may still be underestimated, with only 15% of symptomatic patients receiving appropriate treatment [183]. OSA is also related to numerous common eye diseases that have the potential to reduce vision and overall quality of life. Some of these ocular complications, such as CSC and DME, are reversible; however, they can cause permanent visual impairment if they are not treated properly.

Uncontrolled OSA may lead to persistent pathophysiological alterations such as increased oxidative stress, systemic inflammation, and autonomic dysfunction with sympathetic activation, which may exacerbate the associated ocular diseases. Despite some favorable evidence showing the efficacy of CPAP therapy, the standard of care to treat OSA, on several ocular complications (Table 2), particularly for NAION, DR, and DME, its efficacy in other OSA-associated ocular diseases remains controversial. Since serum levels of inflammatory markers (IL-6, IL-8, TNF-α) are significantly reduced after 3 months of CPAP therapy, clinicians may consider it in OSA patients who respond poorly to treatments for the associated eye diseases [82].

## 7. Conclusions

For patients who present with certain eye conditions or who do not respond well to standard treatment options, ophthalmologists can identify missed OSA at an earlier stage to minimize comorbidities of OSA. Understanding these OSA-related ocular complications will also help educate clinicians caring for patients with OSA to schedule ophthalmology consultations when the aforementioned symptoms are present.

Additional large-scale and prospective studies to identify the underlying factors and potential treatment options, providing more evidence, will reveal whether or not CPAP has the true capacity to reverse pathological alterations caused by OSA. Further studies focusing on common patho-mechanisms, such as inflammatory cytokines and oxidative stress, may provide alternative treatment options for patients with OSA suffering from refractory ocular complications.

## Figures and Tables

**Table 1:** Possible common pathophysiology between OSA and associated ocular complications.

● Oxidative stress
● Autonomic dysfunction with sympathetic activation
● Upregulation of systemic inflammation
● Endothelium dysfunction and disruption of the blood–retinal barrier (BRB)
● Dysregulation of circadian genes

**Table 2:** Ocular complications that may benefit from CPAP treatment.

● Non-arteritic anterior ischemic optic neuropathy (NAION)
● Thyroid eye disease (TED)
● Floppy eyelid syndrome (FES)
● Central serous chorioretinopathy (CSC)
● Diabetic retinopathy (DR)
● Diabetic macular edema (DME)

## Data Availability

Not applicable.

## Abbreviations

AHI	Apnea–Hypopnea Index
BMI	body mass index
BRB	blood–retinal barrier
CI	confidence interval
CLOCK	circadian locomotor output cycles kaput
CPAP	continuous positive airway pressure
CRP	C-reactive protein
CRVO	central retinal vein occlusion
CSA	central sleep apnea
CSC	central serous chorioretinopathy
DES	dry eye syndrome
DM	diabetes mellitus
DME	diabetic macular edema
DR	diabetic retinopathy
ECM	extracellular matrix
FES	floppy eyelid syndrome
fT3	free triiodothyronine
HR	hazard ratio
ICAM	intercellular adhesion molecule
IL	interleukin
IRR	incidence rate ratio
IOP	intraocular pressure
KC	keratoconus
MMP9	matrix metalloproteinase 9
NAION	non-arteritic anterior ischemic optic neuropathy
NPDR	non-proliferative diabetic retinopathy
NTG	normal tension glaucoma
ODI	oxygen desaturation index
OR	odds ratio
OSA	obstructive sleep apnea
PDR	proliferative diabetic retinopathy
PSG	polysomnography
RNFL	retinal nerve fiber layer
RVO	retinal vein occlusion
SA	sleep apnea
TBUT	tear break-up time
TED	thyroid eye disease
TNF-α	tumor necrosis factor-alpha
TSH	thyroid-stimulating hormone
VCAM	vascular cell adhesion molecule
VEGF	vascular endothelial growth factor
VEP	visual evoked potential

## Appendix A

**Table A1:** Summary of the major studies evaluating the association between OSA and glaucoma.

Author, Year	Country/Region	Sample Size	Main Findings
Tsang, 2006 [45]	China	76	Higher AHI in OSA patients was linked to a higher incidence of visual field defect and glaucomatous optic nerve changes.
Sergi, 2007 [32]	Italy	91	The degree of OSA was correlated with IOP, visual field defect, and the mean thickness of the retinal nerve fiber layer (*p* < 0.01 to 0.001). OSA may contribute to the development of NTG.
Kiekens, 2008 [58]	Belgium	21	Higher diurnal variations of IOP occurred in OSA patients after CPAP therapy, which may have an effect on glaucoma progression.
Stein, 2011 [34]	USA	2,182,315; 2,255,863 ^a^	The risk of open-angle glaucoma/NTG did not differ between those with and without SA treated with CPAP and those without SA.
Boyle-Walker, 2011 [31]	USA	70,960	Patients with OSA were more likely to have a diagnosis of glaucoma (OR (95% CI): 1.736 (1.509–1.996), *p* < 0.001).
Lin, 2013 [30]	Taiwan	7084	A large-scale retrospective matched-cohort study showed an increased risk of open-angle glaucoma in subjects during a 5 year period of follow-up after a diagnosis of OSA (HR (95% CI): 1.67 (1.30–2.17), *p*<0.001).
Adam, 2013 [36]	Turkey	83	There was no difference in RNFL thickness, and no significant difference between AHI and IOP in OSA subjects and controls was noted.
Cohen, 2015 [59]	Israel	51	Patients with OSA had significantly higher nocturnal IOP, while CPAP treatment did not cause an increase in IOP.
Huon, 2016 [37]	USA	2,270,918	A meta-analysis of 10 cross-sectional and 2 case–control studies analyzing the prevalence of glaucoma among patients with/without OSA revealed increased odds of glaucoma diagnosis in OSA patients (pooled OR (95% CI): 1.242 (1.204–1.277), *p* < 0.001).
Huon, 2016 [37]	USAs	8244	A meta-analysis of 6 case–control studies analyzing the prevalence of OSA among patients with/without glaucoma revealed increased odds of OSA diagnosis in glaucoma patients (pooled OR (95% CI): 1.746 (1.230–2.477), *p* = 0.002).
Wang, 2017 [39]	China	1757	A systematic review and meta-analysis of 17 studies revealed significantly thinner RNFL in all quadrants in OSA patients than in the control subjects.
Lee, 2019 [40]	Australia	848	Patients with OSA had significantly thinner parapapillary RNFL, which was associated with higher AHI.
Kara, 2018 [41]	Turkey	195	There was a significant negative correlation between the severity of OSA and the thickness of the ganglion cell layer.
Abdullayev, 2019 [42]	Turkey	59	The ganglion cell complex was significantly thinner in OSA patients than in the controls.
Ucak, 2020 [44]	Turkey	104	Severe OSA was significantly associated with higher IOP values (*p* = 0.001) and lower average RNFL thickness (*p* = 0.001).
Lin, 2020 [60]	Taiwan	32	A prospective study showed a significant improvement in visual sensitivity, RNFL thickness, and macular layer thickness in OSA patients after 3 months of CPAP treatment with good adherence.

a. 2,182,315 for open-angle glaucoma analysis; 2,255,863 for NTG analysis.

**Table A2:** Summary of the major studies evaluating the association between OSA and ischemic optic neuropathy.

Author, Year	Country/Region	Sample Size	Main Findings
Stein, 2011 [34]	USA	2,257,163	OSA patients without CPAP therapy had a significantly increased risk of NAION (HR (95% CI): 1.16 (1.01–1.33), *p* < 0.001). There was no significant difference in the risk of NAION between OSA patients treated with CPAP and non-OSA patients.
Huon, 2016 [37]	USA	274	A meta-analysis of 4 case–control studies showed that OSA was significantly associated with the development of NAION (pooled OR (95% CI): 3.126 (1.799–5.433), *p* < 0.001).
Wu, 2016 [37]	China	5,916	A meta-analysis of 4 prospective cohort studies and 1 case–control study demonstrated higher incidence of NAION in OSA individuals vs. non-OSA controls (pooled OR (95% CI): 6.18 (2.00–19.11), *p* = 0.002).
Liguori, 2018 [67]	Italy	20	Stringently adherent CPAP therapy significantly improved VEP in patients with OSA.
Sun, 2019 [63]	Taiwan	42,440	OSA was a significant risk factor for the development of NAION (HR (95% CI): 1.66 (1.08–2.55), *p* = 0.019).
Chang, 2019 [66]	USA	318	Poor compliance with CPAP therapy in patients with moderate-to-severe OSA was a major risk factor of NAION in the fellow eye (HR (95% CI): 4.50 (1.79–11.3), *p* = 0.0015).

**Table A3:** Summary of the major studies evaluating the association between OSA and TED.

Author, Year	Country/Region	Sample Size	Main Findings
Bruyneel, 2019 [74]	Belgium	280	A higher prevalence of thyroid disorders (16.4%) in the OSA population as compared to the general population, particularly in patients with higher BMI values, was noted.
Bozkurt, 2012 [75]	Turkey	245	OSA patients presented with higher prevalence of Hashimoto’s thyroiditis in conjunction with higher OSA severity, especially among women.
Habib, 2019 [78]	USA	109	The prevalence of higher-risk OSA in patients with compressive optic neuropathy from TED was significantly higher than those without TED-related compressive optic neuropathy (59.2% vs. 32.8%, *p* = 0.006).
Godfrey, 2020 [79]	USA	85	A prospective observational cohort study revealed that the risk of compressive optic neuropathy (*p* = 0.014) and vertical strabismus (*p* = 0.047) from TED was significantly higher in patients with a higher risk of OSA.
Petrone, 2016 [80]	Italy	185	Normalization of fT3 was noted after CPAP treatment in OSA patients with euthyroid sick syndrome.
Meston, 2003 [81]	UK	101	TSH was decreased after CPAP treatment (*p* ≤ 0.03) without changes in free T4 level.

**Table A4:** Summary of the major studies evaluating the association between OSA and RVO.

Author, Year	Country/Region	Sample Size	Main Findings
Chou, 2012 [131]	Taiwan	35,634	SA was an independent risk factor for RVO, bringing a 1.94-fold increase in the incidence of RVO after adjusting for age, sex, and comorbidities (95% CI: 1.03 to 3.65, *p* = 0.041).
Agard, 2018 [132]	France	114	A prospective study revealed a significantly higher prevalence of OSA in RVO patients compared to the controls (adjusted OR (95% CI): 5.65 (1.60–19.92), *p* = 0.007), although no significant relationship between the degree of OSA and RVO was noted.
Wang, 2019 [133]	China	60	Mean AHI and ODI were significantly higher in patients with CRVO (*p* = 0.008 and 0.001, respectively). A positive correlation was identified between AHI and BMI (*r* = 0.476, *p* = 0.017).
Brent, 2020 [134]	Canada	27	Screening of 27 newly diagnosed RVO patients using PSG revealed a higher prevalence (up to 96%) of severe OSA. Mandatory OSA screening was recommended for all patients with RVO.
Wan, 2021 [135]	China	90	The oxidative stress factors and inflammatory biomarkers were elevated in serum from patients with OSA and RVO, and serum levels of oxidative/inflammatory biomarkers were positively correlated with the severity of OSA. OSA was a negative prognostic factor of visual activity improvement in patients with RVO.

**Table A5:** Summary of the major studies evaluating the association between OSA and CSC.

Author, Year	Country/Region	Sample Size	Main Findings
Huon, 2016 [37]	USA	156	A meta-analysis of 2 studies revealed a significantly increased odds of OSA in patients with CSC (pooled OR (95% CI): 2.019 (1.078–3.781), *p* = 0.028).
Wu, 2018 [139]	USA	7238	A systematic review and meta-analysis of 6 studies found that patients with CSC had a higher risk for OSA (OR (95% CI): 1.56 (1.16–2.10)).
Brodie, 2015 [140]	USA	98	Patients with CSC did not have significantly higher rates of OSA risk or previous diagnosis than age/gender/BMI-matched controls.
Chatziralli, 2017 [141]	Greece	366	OSA conferred a significantly higher risk for developing CSC (OR (95% CI): 4.97 (1.40–17.60)).
Liu, 2020 [3]	Taiwan	333,343	Patients with SA had a significantly increased incidence rate of CSC compared to age- and sex-matched controls (IRR (95% CI): 1.2 (1.1–1.4), *p* < 0.0001) after adjusting for sex, age, residency, income level, and comorbidities. SA patients receiving CPAP titration had a significantly lower incidence rate of CSC than the other patients (IRR (95% CI): 0.5 (0.4–0.6), *p* < 0.0001).
Pan, 2020 [142]	USA	15,250,959	The incidence of CSC was higher in patients with OSA (HR: 1.081, *p* < 0.033). OSA was associated with an increased risk of CSC in both genders.

**Table A6:** Summary of the major studies evaluating the association between OSA and DR.

Author, Year	Country/Region	Sample Size	Main Findings
Subramanian, 2019 [163]	United Kingdom	1,656,739	The adjusted IRR of OSA in patients with type 2 DM was 1.48 (95% CI: 1.42 to 1.55; *p* < 0.001) compared to patients without type 2 DM. Obesity, male sex, and excessive BMI were the strongest predicting factors for OSA in individuals with type 2 DME (OR: 8.29, 2.27, and 2.02, respectively).
Laaban, 2009 [164]	France	303	The prevalence of DR did not significantly correlate to the severity of OSA.
Banerjee, 2013 [165]	Australia	93	The presence of OSA and concomitant hypoxia was not associated with the development of diabetic retinal complications.
Kosseifi, 2010 [166]	USA	98	The frequency of DR was increased with the severity of OSA, even in well-controlled type 2 DM.
Chang, 2018 [167]	USA	317	Patients with severe OSA were at increased risk of developing DR (OR (95% CI): 2.18 (1.14–4.18), *p* = 0.019) and proliferative DR (OR: 2.40 (1.12–5.0), *p* = 0.024).
Chew, 2020 [168]	Singapore	92	Vision-threatening DR was associated with a higher AHI (OR (95% CI): 1.06 (1.02–1.10)) and higher risk of moderate OSA (OR: 4.73 (1.46–15.31)).
Mori, 2019 [169]	Japan	131	The severity of OSA by AHI was independently associated with the development of DR.
Shiba, 2019 [170]	Japan	132	The 4% oxygen saturation index (4% oxygen desaturation events per hour) was significantly higher in the PDR group than in the NPDR group.
Smith, 2019 [178]	USA	321	Patients with OSA who were compliant with CPAP therapy had a significantly lower risk of developing DR as compared to noncompliers (OR (95% CI): 0.54 (0.31–0.94), *p* = 0.04).

**Table A7:** Summary of the major studies evaluating the association between OSA and DME.

Author, Year	Country/Region	Sample Size	Main Findings
Chang, 2018 [167]	USA	317	Risk of DME was significantly higher in patients with severe OSA (OR (95% CI): 2.89 (1.58–5.27), *p*= 0.001).
Dot, 2019 [173]	France	99	Severe OSA with AHI over 30 and higher cumulative time of SpO_2_ less than 90% were associated with an increased risk of developing DME
Nesmith, 2014 [174]		30	Patients who responded poorly to anti-VEGF therapy for DME were at a significantly higher risk of having OSA.
Chiang, 2021 [175]	Taiwan	54	Severe OSA was a risk factor for DME (OR (95% CI): 7.36 (1.32–40.96), *p* = 0.023). Severe OSA patients had a higher frequency of refractory DME than non-severe OSA patients (*p* = 0.009).
Mason, 2012 [177]	UK	35	A possible association between CPAP usage and improvement in visual acuity after 6 months of treatment was noted in patients with DME and OSA.
West, 2018 [179]	UK	131	A prospective randomized controlled trial of patients with DME and OSA demonstrated no increase in visual function gains at 12 months with CPAP for OSA compared to controls.
Li, 2021 [175]	Taiwan	14,152	A retrospective clinical cohort study showed that severity of OSA is a risk factor of DME (HR (95% CI): 2.97 (1.08–8.16), *p* = 0.034) and is associated with developing refractory DME.
